# Metastatic small cell neuroendocrine carcinoma of the submandibular gland from the lung: A case report

**DOI:** 10.1097/MD.0000000000019018

**Published:** 2020-01-24

**Authors:** Dong Hoon Lee, Jo Heon Kim, Tae Mi Yoon, Joon Kyoo Lee, Sang Chul Lim

**Affiliations:** a Department of Otolaryngology-Head and Neck Surgery; b Department of Pathology, Chonnam National University Medical School & Hwasun Hospital, Hwasun, South Korea.

**Keywords:** lung neoplasms, neoplasm metastasis, neuroendocrine tumors, small cell carcinoma, submandibular gland

## Abstract

**Rationale:**

Small cell neuroendocrine carcinoma of the salivary gland is an extremely rare condition. To the best of our knowledge, metastasis of small cell neuroendocrine lung cancer to the submandibular gland has not been reported in the literature.

**Patient concern:**

An 87-year-old female complained of a left neck mass that enlarged from one month ago.

**Diagnosis:**

The final diagnosis was diagnosed as a metastatic small cell neuroendocrine carcinoma of the submandibular gland from lung by an immunohistochemistry.

**Interventions:**

Left submandibular resection was performed under general anesthesia.

**Outcomes:**

We recommended further evaluation and treatment, but the patient and patient family support team rejected further treatment of her condition. It was confirmed that 3 months after this conclusive diagnosis, the patient died as a result of this condition and disease.

**Lessons:**

Small cell neuroendocrine carcinoma of the salivary gland is an extremely rare condition. We report a case of metastatic small cell neuroendocrine carcinoma of the submandibular gland from the lung.

## 1 Introduction

Small cell neuroendocrine carcinoma arises most commonly in the lung region of the body, however the occurrence of this condition in the salivary gland is considered to be extremely rare.^[1–8]^ Small cell neuroendocrine lung carcinoma mainly metastasizes to the liver, adrenals, bones or brain.^[9]^ To the best of our knowledge, metastasis of small cell neuroendocrine lung cancer to the submandibular gland has not been reported in the literature. Herein, we report a case of metastatic small cell neuroendocrine carcinoma of the submandibular gland as noted from lung cancer.

## 2 Case report

**Figure 1:**
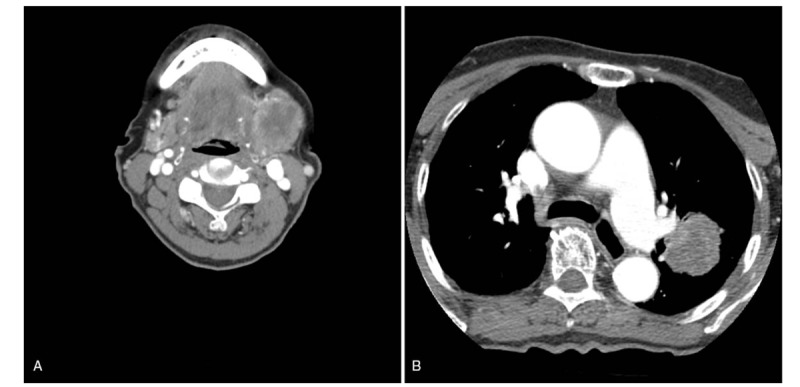
CT scans demonstrate a 3.7 × 3.1 cm sized heterogeneous enhanced, central necrotic lesion with irregular margin in the left submandibular gland (A), and approximately 3.7 × 3.2 cm sized mass in the left upper lobe (B).

An 87-year-old female was referred for a 1-month history of an enlarging left neck mass. Upon a physical examination it was revealed the presence of a 3 cm sized left submandibular mass. The facial nerve function was noted to be intact. The Computed tomography (CT) scans as taken at a local hospital revealed a 3.7 × 3.1 cm sized heterogeneous enhanced, central necrotic lesion with an irregular margin in the left submandibular gland (Fig. 1). On CT scan, approximately 3.7 × 3.2 cm sized mass was also detected in the left upper lobe (Fig. 1). However, there was no cervical or mediastinal lymphadenopathy as noted at that time.

**Figure 2:**
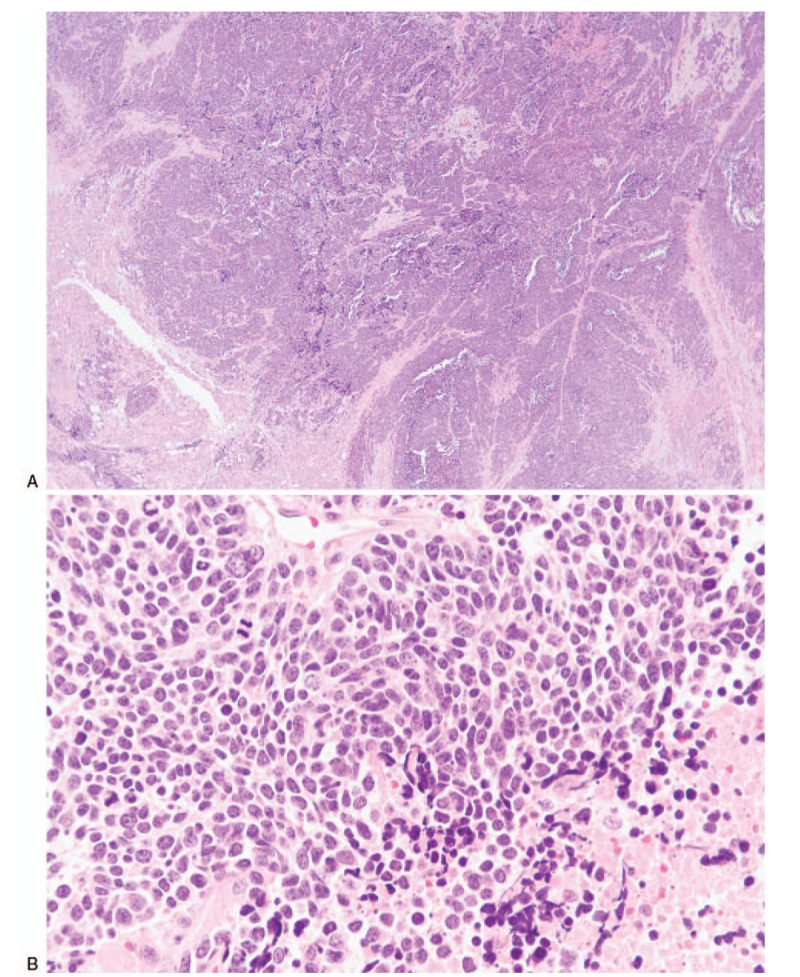
(A) Hypercellular tumor infiltrates the salivary parenchyma (lower left). Growth patterns include diffuse or trabecular (Hematoxylin and eosin stain, ×10). (B) Small cells with dispersed nuclear chromatin, inconspicuous nucleoli, minimal cytoplasm, high mitotic rates and foci of coagulative necrosis (lower right) are present (Hematoxylin and eosin stain, ×400).

The preoperative diagnosis of this patient was thought to be the metastasis of lung cancer to the submandibular gland. In fact, a left submandibular resection was performed under general anesthesia 2 days after 1st visit, to determine the diagnosis and treatment direction best suited for the patient. The submandibular tumor was relatively well separated from surrounding tissues and therefore had no involvement of the marginal mandibular nerve. The histopathologic examination of left submandibular mass was diagnosed as small cell neuroendocrine carcinoma metastases noted from lung cancer (Fig. 2). The mass was strongly positive for CD56, synaptophysin, CK, and TFT-1 on immunohistochemical staining. The clinical stage of this patient was T2N0M1. We recommended further evaluation and treatment, but the patient and patient family support team rejected further treatment of her condition. It was confirmed that 3 months after this conclusive diagnosis, the patient died as a result of this condition and disease. Patient has provided informed consent for publication of the case.

## 3 Discussion

Generally speaking, a small cell carcinoma can occur in any part of the body, but it is shown to mainly occur in the region of the lungs. In this sense, the small cell neuroendocrine carcinoma of the salivary gland is an extremely rare conditon.^[1–8]^ In the salivary gland, the parotid gland was the most common occurrence site where this disease is found, followed by the submandibular gland.^[1,2]^ The rarity of a small cell neuroendocrine carcinoma in salivary glands is more than likely associated with the absence of the neuroendocrine cells in the salivary glands.^[5]^

Small cell neuroendocrine lung carcinoma mainly metastasizes to the liver, adrenals, bones or brain.^[9]^ There has been no report of small cell neuroendocrine lung carcinoma metastases to the submandibular gland, as best reviewed to our knowledge.

The presenting symptom of small cell neuroendocrine carcinoma of the submandibular gland is characteristically seen as an asymptomatic, palpable mass.^[1–4]^ The other symptoms of this condition in a patient include pain, paresthesia, paraneoplastic syndrome, and facial nerve palsy.^[1,3]^ In our case, the patient presented only with a palpable mass without pain or tenderness, as located in the left submandibular area.

The final diagnosis of a small cell neuroendocrine carcinoma of the submandibular gland is only possible by the use of a histopathologic examination, especially noting an immunohistochemistry.^[1–8]^ Immunohistochemistry of an small cell neuroendocrine carcinoma shows diffuse cytoplasmic staining with CD56, chromogranin, and synaptophysin.^[1–3]^ The entire body needs to be examined to distinguish between the incidence of a primary and metastatic small cell neuroendocrine carcinoma of the submandibular gland.^[4]^ Chest or abdomen CT, brain magnetic resonance imaging (MRI), and positron emission tomography (PET) are performed to confirm metastasis and stage.^[9–11]^ In our case, the patient and her families refused the examinations for the presence of a lung lesion, therefore, we could not rule out the possibility of separate cancers also being present. However, the mass that was present was clearly seen, and it was diagnosed as a metastatic small cell neuroendocrine carcinoma of the submandibular gland from lung by an immunohistochemistry. The differential diagnosis of a small cell neuroendocrine carcinoma of the submandibular gland includes a malignant lymphoma, embryonal rhabdomyosarcoma, Ewing's sarcoma, malignant melanoma, and neuroblastoma.^[1–4,6]^

The treatment of small cell neuroendocrine carcinoma of the submandibular gland has not been established because of the rarity of the condition.^[1–4,6,8]^ In localized small cell neuroendocrine carcinoma of the submandibular gland, surgical excision with postoperative chemotherapy or radiation therapy can usually be counted on an effective treatment.^[1,4,5]^ However, chemotherapy is the first treatment for metastatic cancer, like with most of our patients.^[2,4,8]^

The primary small cell neuroendocrine carcinoma of the submandibular gland had a better prognosis than occurs in other primary sites.^[1–4,6]^ However, these carcinomas have an aggressive behavior and are well known to cause recurrence and regional and distant metastasis.^[1–4,6]^ The median overall survival for patients with metastatic small cell lung carcinoma receiving standard chemotherapy has remained in range of 9 to 11 months.^[9]^ The risky prognostic factors include old age, carcinomas larger than 3 cm, presence of distant metastasis, and CK20-negative tumors.^[2]^ Our patient, who had several high risk prognostic factors, died within 3 months of the diagnosis.

In conclusion, small cell neuroendocrine carcinoma of the salivary gland is an extremely rare condition. Moreover, the metastasis of small cell neuroendocrine lung cancer to the submandibular gland has not been reported as reviewed to the best of our knowledge.

## Author contributions

**Conceptualization:** Dong Hoon Lee.

**Data curation:** Dong Hoon Lee, Jo Heon Kim, Tae Mi Yoon, Joon Kyoo Lee, Sang Chul Lim.

**Investigation:** Dong Hoon Lee.

**Writing – original draft:** Dong Hoon Lee.

**Writing – review & editing:** Dong Hoon Lee, Jo Heon Kim, Tae Mi Yoon, Joon Kyoo Lee, Sang Chul Lim.
